# Physiotherapist-guided, wearable-informed exercise improves 6-minute walk distance in patients with type 2 diabetes, including those with diabetic kidney disease: a prospective study

**DOI:** 10.1007/s13340-026-00900-x

**Published:** 2026-04-22

**Authors:** Momo Takahashi, Yuma Tamura, Hajime Tamiya, Masato Terashima, Harunori Takahashi, Nobuyuki Banba, Yuki Nakatani, Naoyuki Otani, Asuka Ueno, Takanori Yasu, Yasuhiro Maejima

**Affiliations:** 1 Department of Rehabilitation, Dokkyo Medical University Nikko Medical Center, 145-1 Moritomo Nikko, Tochigi, 321-1298 Japan; 2 Niigata University of Health and Welfare, 1398 Shimami-cho, Kita-ku, Niigata, 950-3198 Japan; 3 Department of Rehabilitation, Dokkyo Medical University Saitama Medical Center, 2-1-50 Minamikoshigaya, Koshigaya, Saitama 343-8555 Japan; 4 Department of Diabetes and Endocrinology, Dokkyo Medical University Nikko Medical Center, 145-1 Moritomo Nikko, Tochigi, 321-1298 Japan; 5 Department of Cardiology, Dokkyo Medical University Nikko Medical Center, 145-1 Moritomo Nikko, Tochigi, 321-1298 Japan; 6 Department of Cardiovascular Medicine and Nephrology, Dokkyo Medical University Nikko Medical Center, 145-1 Moritomo Nikko, Tochigi, 321-1298 Japan

**Keywords:** Wearable device, Diabetes mellitus, Kidney disease, Physiotherapist, 6-min walk distance

## Abstract

**Background:**

Evidence for wearable-guided physiotherapy in diabetic kidney disease (DKD) is limited. This study evaluated the effects of physiotherapist-supervised exercise supported by wearable device feedback in patients with type 2 diabetes, including those with DKD.

**Methods:**

In this single-center, prospective study, 58 outpatients were allocated to an intervention group (IG) or non-intervention group (NIG). The per-protocol set included 45 participants completing six months (IG = 25, NIG = 20). IG wore a wrist-worn device and received monthly physiotherapist-led guidance; NIG received standard care. The primary endpoint was 6-min walk distance (6MWD); secondary outcomes included weight-bearing index (WBI), phase angle (PhA), HbA1c, and eGFR. Analyses used linear mixed-effects models adjusted for covariates.

**Results:**

IG improved 6MWD from 512.0 m to 551.0 m (+ 38.7 m), while NIG changed from 410.0 m to 407.0 m (− 3.7 m). The group × time interaction was significant (*p* < 0.001); adjusted between-group difference was + 42.4 m (95% CI: 20.3–64.5). IG showed greater reductions in HbA1c (− 0.89%, 95% CI: − 1.53 to − 0.25; *p* = 0.008) and improvements in WBI and PhA (*p* < 0.01). Changes in eGFR were not significant (0.65 mL/min/1.73m^2^, 95% CI: -3.84 to 5.14; *p* = 0.78).

**Conclusions:**

Monthly physiotherapist-led exercise supported by wearable feedback improved functional capacity and glycemic control in patients with DKD. This pragmatic, scalable model may enhance outpatient rehabilitation strategies.

**Graphical abstract:**

**Figure:**
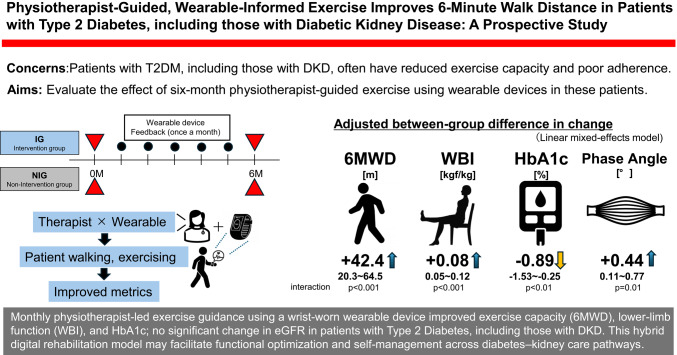

Values represent adjusted between-group differences in change from baseline to six months, estimated using linear mixed-effects models with group, time, and group × time interaction, adjusted for age, baseline 6-min walk distance, HbA1c, eGFR, cardiovascular disease, and smoking status. Error ranges indicate 95% confidence intervals. Arrows indicate direction of change favoring the intervention group.

**Supplementary Information:**

The online version contains supplementary material available at 10.1007/s13340-026-00900-x.

## Introduction

Diabetic kidney disease (DKD) is a major microvascular complication of diabetes and a leading cause of chronic kidney disease (CKD) progression and dialysis [1, 2]. It also increases cardiovascular and all-cause mortality risk, highlighting the need to prevent its progression.

Exercise therapy improves glucose metabolism, insulin sensitivity, and cardiovascular health in diabetes [3, 4], and moderate-intensity exercise benefits patients with CKD by maintaining physical function and quality of life [5, 6]. Six-minute walk distance (6MWD) correlates with maximal oxygen uptake in diabetes [7], and resistance exercise improves 6MWD by approximately 90 m in CKD [8]. The weight-bearing index (WBI), indicating muscle strength relative to body weight, also increases significantly with exercise [9]. Supervised exercise programs are particularly effective in improving capacity and strength [10]. The clinical efficacy of physiotherapist-led exercise programmes is well established in patients with type 2 diabetes and chronic kidney disease (CKD). Notably, our institution's prior research demonstrated that tailor-made exercise intervention reduces the risk of developing cardiovascular diseases and all-cause mortality [11]. These findings provide an important clinical foundation for exercise-based rehabilitation in this population.

However, physiotherapist involvement is not mandatory for calculating the Diabetes Dialysis Prevention Guidance and Management (including Guidance of Patients with Severe Renal Impairment). A national survey by the Japanese Society of Nephrology reported that only around 13% of physiotherapists were involved in the calculation process [12]. As physicians frequently lack time for detailed exercise guidance and many patients are ineligible for rehabilitation reimbursement, adherence to exercise therapy is often poor due to low self-efficacy [13, 14].

Wearable device–based interventions have recently shown promise in improving self-management, medication adherence, and glycemic control [15]. Moreover, wearable-guided rehabilitation enhances activity and cardiovascular outcomes in heart disease [16]. Building on the established effectiveness of physiotherapist-led exercise guidance, the integration of wearable device–informed feedback may offer a pragmatic strategy to support exercise implementation in routine outpatient care by enhancing self-monitoring, motivation, and continuity of guidance between supervised sessions.

Therefore, this study evaluated the effects of a wrist-worn wearable device-based, physiotherapist-supervised exercise intervention on exercise capacity, physical function, renal outcomes, and metabolic parameters in patients with type 2 diabetes, including those with DKD.

## Materials and methods

### Study design and participants

This was a single-center, prospective, non-randomized intervention study. This study was registered with the University Hospital Medical Information Network Clinical Trials Registry (UMIN-CTR; ID: UMIN000059381). The primary outcome was the 6MWD. Participants were recruited between September 2022 and September 2023, and the 6-month intervention and follow-up period was completed by March 2024.

Sample size was estimated using G*Power 3.1 for the group × time interaction in a two-group, two-time-point measures design, informed by prior exercise intervention studies in CKD and our preliminary institutional data [17]. An effect size of f = 0.25 was assumed, corresponding to a mean 6MWD improvement of approximately 35–45 m with an SD of change of ~ 65 m. Additionally, a correlation between time points of r = 0.5 was assumed and incorporated as a parameter for the sample size estimation. With α = 0.05 and 80% power, the required sample size was 36 participants; allowing for 20% attrition, a target of 45–50 participants was set.

**Fig. 1:**
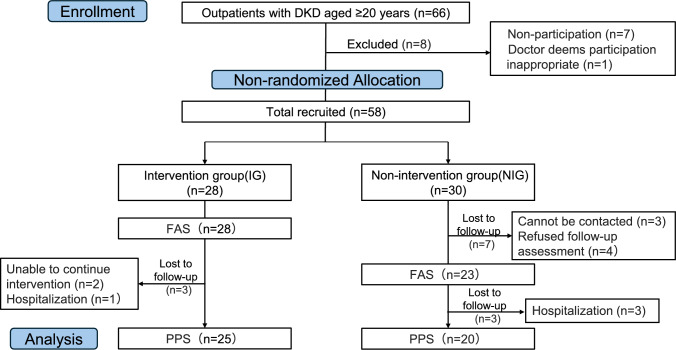
Study flowchart Flow diagram of participant recruitment, allocation, and analysis. A total of 66 outpatients with diabetic kidney disease (DKD) aged ≥ 20 years were screened. Twenty-eight participants were allocated to the intervention group (IG) and 30 to the non-intervention group (NIG). Exclusions were due to non-participation (n = 7) and ineligibility determined by the physician (n = 1). In the IG, no patient was excluded, resulting in 28 patients in the Full analysis set (FAS). In the NIG, four patient withdrew consent and three were excluded due to loss of contact, resulting in 23 patients in the FAS. After further exclusions and dropouts (three in the IG and three in the NIG), 25 participants in the IG and 20 in the NIG completed the 6-month follow-up and were included in the per-protocol set (PPS). ITT analysis was not performed because all outcomes at six months were completely missing for dropouts. FAS included all participants who completed baseline assessment

Overall, 58 outpatients with DKD aged ≥ 20 years were screened, and all provided written informed consent (Fig. 1). The exclusion criteria were as follows: (1) ongoing hemodialysis, (2) acute coronary syndrome or stroke within the preceding three months, (3) inability to provide informed consent, and (4) any other contraindications to exercise as specified in current cardiac rehabilitation guidelines. Thirteen participants did not undergo the 6-month assessment due to hospitalization (n = 4), new orthopedic conditions (n = 2), loss to follow-up (n = 3), or refusal of the final assessment after learning its time and physical demands (n = 4). Finally, 45 participants completed all evaluations per the per-protocol set (PPS), and their data were included in the final analysis. Attending physicians were instructed not to change participants’ medications during the study period.

### Study protocol

**Fig. 2:**
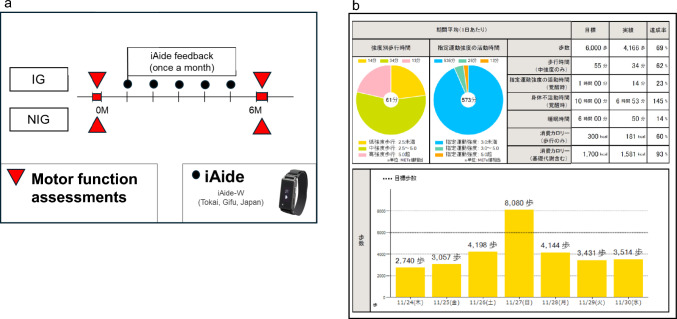
Study protocol (a) and feedback forms for the iAide (b) (a) Study protocol. The iAide-W was worn on the wrist only in the intervention group (IG). Each IG participant received monthly, face-to-face feedback from a trained physical therapist based on their own iAide data. (b) The iAide report form*.* Continuous 24-h step count and activity data were collected using the iAide, worn on the wrist in the IG. Face-to-face feedback was provided monthly over the 6-month study period. The top panel of the report displays average weekly walking time and activity time by intensity category. The second, third, and bottom panels display the number of steps, walking time by intensity, and daily activity time by intensity, respectively

All patients were asked whether they would be willing to wear a wrist-worn wearable device (wearable device) and receive one face-to-face session per month with a physiotherapist; those who agreed were assigned to the intervention group (IG). Participants who were unable to attend monthly physiotherapy sessions due to scheduling difficulties or time constraints were assigned to the NIG and continued to receive usual medical care (Fig. 1). The fitting schedule for the wearable device is shown in Fig. 2A. At baseline and six months, both groups underwent motor function assessments. However, feedback was provided only to the IG.

## Measurement outcomes

### Main outcome

The 6MWD was measured as the total distance walked (m) according to the American Thoracic Society standard protocol [18].

## Secondary outcome

### Motor function

Isometric knee extension muscle strength was measured using a pull sensor for manual muscle testing (MT-150, SAKAIMED). The WBI was defined as maximal isometric knee extension force normalized to body weight (kgf/kg). Measurements were performed twice for each leg, and the highest recorded value was adopted as the muscle strength value for analysis. Test–retest reliability of WBI measurements has been reported to be high in previous studies (intraclass correlation coefficient > 0.90) [19, 20].

### Laboratory data

Laboratory data included eGFR, urinary albumin/creatinine ratio (UACR), HbA1c, and lipid profile at the start of the observation period. DKD severity was classified according to eGFR and UACR [21]. These data were collected from outpatient examinations, blood tests, and urinalyses. Furthermore, the nephropathy stage was classified using eGFR and the UACR [20]. As an indicator of daily estimated salt intake, salt intake was calculated from sodium (Na) and Cr levels in spot urine samples at the outpatient visit [22, 23].

Estimated daily salt intake (g/day) was calculated using the Tanaka formula:

$$ \begin{gathered} Saltintake(g/day) = \hfill \\ 21.98 \times (Na/Cr)^{{0.392}} \times \frac{{Predicted\;24 - h\;urinary\;creatinine\;excretion\;(mg/day)}}{{17.1}} \hfill \\ \end{gathered} $$

The predicted 24-h urinary creatinine excretion was estimated using sex-specific equations:

$$ \begin{gathered} {\mathrm{Men}}:{\text{ 14}}.{\text{89 }} \times {\text{body weight }}\left( {{\mathrm{kg}}} \right){\text{ }} + {\text{ 16}}.{\text{14 }} \hfill \\ \times {\text{height }}\left( {{\mathrm{cm}}} \right){\text{ }} - {\text{ 2}}.0{\text{4 }} \times {\text{ age }}\left( {{\mathrm{years}}} \right){\text{ }} - {\text{ 2244}}.{\mathrm{45}} \hfill \\ \end{gathered} $$

$$ \begin{gathered} {\mathrm{Women}}:{\text{ 8}}.{\text{58 }} \times {\text{body weight }}\left( {{\mathrm{kg}}} \right){\text{ }} + {\text{ 5}}.0{\mathrm{9}} \hfill \\ {\text{ }} \times {\text{height }}\left( {{\mathrm{cm}}} \right){\text{ }} - {\text{ 4}}.{\text{72 }} \times {\text{age }}\left( {{\mathrm{years}}} \right){\text{ }} - {\text{ 74}}.{\text{95 }} \hfill \\ \end{gathered} $$

### Bioelectrical impedance analysis (BIA)

Skeletal Muscle Index (SMI) was calculated by summing the amount of skeletal muscle in the limbs/height (m^2^) [24]. Body fat percentage (body fat kg/body weight kg × 100) was calculated using a multi-frequency body composition analyzer (TANITA, MC-180). The phase angle (PhA) is a well-known qualitative marker of skeletal muscle [25]. It was calculated using the formula ‘PhA = arctangent (reactance/resistance) × 180°/π’ [25, 26].

### Intervention

**Safety considerations:** Before participation, patients were instructed to discontinue exercise immediately if they experienced symptoms suggestive of hypoglycemia during or before exercise, and to take appropriate corrective measures (e.g., glucose intake). This precaution was emphasized to prevent adverse events and ensure patient safety throughout the intervention. Participants were also instructed to promptly report any other adverse events, including falls, musculoskeletal symptoms, or cardiovascular symptoms, to the study staff.

Each patient in the IG received monthly face-to-face feedback from a trained physical therapist based on their wearable device data, adhering to the FITT principles (frequency, intensity, time, type of exercise). Activities of daily living were monitored and feedback provided through continuous 6-month wear of the wrist-worn iAide (Tokai Corp., Gifu, Japan (Fig. 2B). The device is an accelerometer that continuously records heart rate, step count and estimated metabolic equivalents (METs), and automatically transmits the data to a secure cloud server. Each patient in the IG received monthly face-to-face feedback from a trained physiotherapist based on data obtained from the wrist-worn wearable device (iAide). The device continuously recorded step counts, heart rate, and estimated metabolic equivalents (METs), which were transmitted to a secure cloud server. Physiotherapists reviewed the uploaded activity data, particularly from unsupervised daily activities, and provided individualized exercise guidance based on daily step counts and MET levels to optimize exercise intensity, frequency, and timing according to the FITT principle. The wearable device was used exclusively as a tool to support physiotherapist-guided feedback during counseling sessions, and detailed longitudinal raw activity data were not retained for subsequent quantitative analysis. Exercise intensity was determined according to the Karvonen formula, with target heart rates set based on nephropathy stage. The exercise guidance was based on our earlier reports [11]. The patients were then instructed to gradually increase by at least 500 steps above the baseline. Targeting an additional 500 steps daily is a common goal of behavioral interventions [27]. Exercise and device adherence were monitored throughout the study, and participants were encouraged to maintain regular exercise and daily device use. The proportion of individuals able to walk at least 6,000 steps per day on ≥ 3 days per week was calculated as the “exercise adherence rate.”

The NIG group served as the control group, receiving standard treatment throughout the 6-month study period, which involved neither wearing a wearable device nor receiving individual exercise guidance from a physiotherapist.

In both groups, standard care consisted of multidisciplinary guidance provided by physicians, nurses, and registered dietitians.

### Statistical analysis

Data are presented as mean ± standard deviation or median with 10th and 90th percentiles for continuous variables, and as numbers and percentages for categorical variables. Baseline comparisons were conducted using Student’s *t*-test or Wilcoxon rank-sum test for continuous variables, and the chi-squared (χ^2^) test or Fisher’s exact test for categorical variables.

Analyses of the study endpoints were performed using a linear mixed-effects model with fixed effects for group (intervention vs. non-intervention), time (baseline vs. six months), and their interaction, and a random intercept for each participant. Prespecified covariates included age, baseline 6MWD, HbA1c, eGFR, cardiovascular disease, and smoking status. For the primary outcome (6MWD), standardized mean differences were calculated and reported as Hedges’ g with corresponding 95% confidence intervals. All secondary outcomes were analyzed in an exploratory manner with emphasis on 95% confidence intervals. As a sensitivity analysis, an intention-to-treat (ITT) analysis including all allocated participants (n = 58) was additionally performed for the primary outcome using the same linear mixed-effects model.

All analyses were conducted using IBM SPSS Statistics version 29 (IBM Corp., Armonk, NY, USA). Statistical significance was set at a two-tailed p-value < 0.05.

## Results

Of the 58 participants initially enrolled, 45 (IG, n = 25; NIG, n = 20) completed the 6-month assessments and were included in the PPS. Baseline characteristics were generally comparable between groups (Table 1).

**Table 1:** Clinical Background of Study Patients (n = 45)

	Non-intervention group	Intervention group	*p*
	n = 20	n = 25	
Age (years)	75.2 ± 10.5	71.3 ± 10.0	0.06
Body mass index (kg/m^2^)	24.5 ± 3.7	26.4 ± 4.8	0.77
Duration of diabetes (month)	162.5 ± 113.7	113.0 ± 77.4	0.34
DKD stage 1, n (%)	9 (45)	7 (28)	0.11
DKD stage 2, n (%)	6 (30)	16 (24)	
DKD stage 3, n (%)	4 (20)	2 (8)	
DKD stage 4, n (%)	1 (5)	0 (0)	
Hypertension, n (%)	7 (28)	9 (45)	0.75
Dyslipidemia, n (%)	7 (28)	9 (45)	0.60
CVD, n (%)	10 (40)	7 (35)	0.73
Current smoker, n (%)	3 (15)	1 (4)	0.31
HbA1c (%)	7.7 ± 0.9	7.3 ± 0.6	0.30
White blood cells (10^2^/μl)	6.21 ± 1.59	6.56 ± 1.24	0.28
Ht (%)	42.5 ± 4.0	44.0 ± 5.1	0.30
T-cho(mg/dL)	162.8 ± 31.4	152.0 ± 35.9	0.28
LDL-C (mg/dL)	91.0 ± 29.9	86.0 ± 33.5	0.44
HDL-C (mg/dL)	48.0 ± 7.4	54.2 ± 12.3	0.18
Triglyceride (mg/dL)	149.5 ± 65.0	110.5 ± 60.1	0.13
eGFR (mL/min/1.73m^2^)	56.1 ± 17.7	58.7 ± 17.5	0.85
Alb/Cre ratio (mg/g・Cre)⁺	#32.0 (5.4, 633.0)	#36.5 (11.8, 246.0)	0.40
Estimated daily salt intake (g/day)	9.8 ± 2.0	9.4 ± 2.0	0.32
Medications			
Insulin, n (%)	0 (0)	2 (8)	0.15
GLP1-RAs, n (%)	2 (8.0)	3 (12)	0.61
Biguanide, n (%)	15 (60)	14 (70)	0.65
DPP4i, n (%)	12 (48)	11 (44)	0.58
SGLT2i, n (%)	13 (50)	15 (55)	0.67
Thiazolidinediones, n (%)	0 (0)	0 (0)	
Sulfonylurea drug, n (%)	1 (4)	2 (10)	0.29
Glinide, n (%)	1 (4)	2 (10)	0.43
AGIs, n (%)	0 (0)	0 (0)	1.00
ARB/ ACEi, n (%)	14 (56)	14 (70)	0.29
β-blocker, n (%)	4 (16)	6 (30)	0.95
Ca channel blocker, n (%)	10 (40)	13 (65)	0.70
Diuretics, n (%)	1 (4)	3 (15)	0.14
ARNI, n (%)	0 (0)	0 (0)	
Anti-platelet, n (%)	12 (46)	10 (40)	0.50

Data are shown as % or mean ± SD or #median (10 percentile, 90 percentile). P values were obtained using Student's t-test, χ2 test, or #Friedman test, as appropriate. Significance levels: p*: *p* < 0.05; **, *p* < 0.01. + Data are shown as median. Abbreviations: ACEi, angiotensin coverting enzyme inhibitor; AGIs, alpha-glucosidase inhibitors; ARB, angiotensin receptor blocker; ARNI, angiotensin receptor-neprilysin inhibitor; DM, diabetes mellitus; HDL-C, high density lipoprotein cholesterol; Ht, hematocrit; IG, intervention group; LDL-C, low density lipoprotein cholesterol; NIG, non-intervention group; T-cho, total cholesterol; SGLT2i, sodium glucose cotransporter 2 inhibitor

**Fig. 3:**
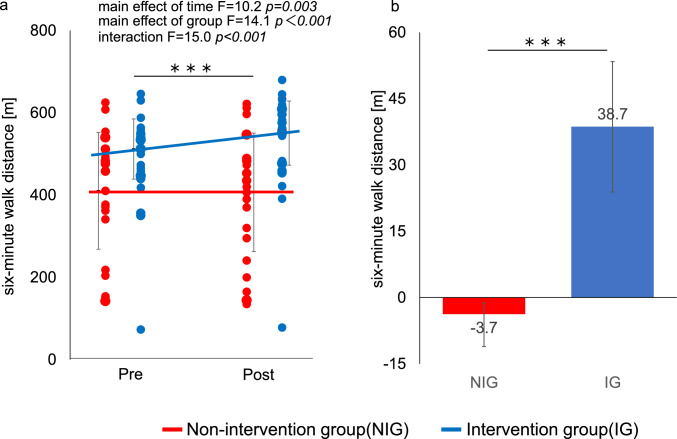
Effects of the intervention on 6-min walk distance (6MWD) (a) Individual values and group means for 6MWD at baseline (Pre) and six months (Post). Points represent individual participants; solid lines indicate group means with error bars showing SD. (b) Mean change in 6MWD from baseline to six months in the intervention group (IG, blue line) and non-intervention group (NIG, red line). Bars represent mean changes with 95% confidence intervals. Between-group differences were assessed using a linear mixed-effects model *p < 0.05, **p < 0.01, ***p < 0.001

**Fig. 4:**
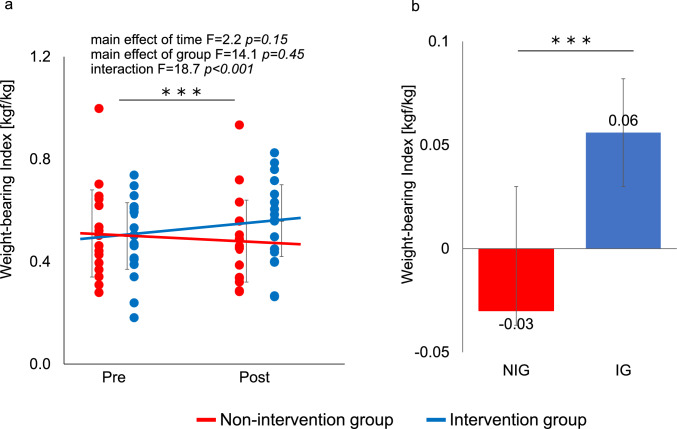
Effects of the intervention on weight bearing Index(WBI) (a) Individual values and group means for WBI at baseline (Pre) and six months (Post). Points represent individual participants; solid lines indicate group means with error bars showing SD. (b) Mean change in WBI from baseline to six months in the intervention group (IG, blue line) and non-intervention group (NIG, red line). Bars represent mean changes with 95% confidence intervals. Between-group differences were assessed using a linear mixed-effects model *p < 0.05, **p < 0.01, ***p < 0.001

In the fully adjusted model using LMM, adjusted for covariates (age, baseline 6MWD, HbA1c, eGFR, cardiovascular disease, smoking status), 6MWD improved significantly in the IG, increasing from 512.0 m to 551.0 m (+ 38.7 m [95% CI, 23.9 to 53.4]), whereas the NIG showed a slight decrease from 410.0 m to 407.0 m (− 3.7 m [95% CI, − 11.1 to − 3.5]). The adjusted between-group difference in change was + 42.4 m [95% CI: 20.3 to 64.5], and the group × time interaction was statistically significant (*p* < 0.001). The standardized effect size (Hedges’ g) was 0.38 (95% CI, 0.21 to 0.97) (Fig. 3a, b). In an additional ITT analysis (full analysis set, n = 58) using the same mixed-effects model, the group × time interaction for 6MWD remained significant (p < 0.001). The adjusted between-group difference in change was + 41.1 m (95% CI 23.5–58.7). Other outcomes were also consistent with the per-protocol analysis (Supplementary Table S1). WBI improved significantly in the IG compared with the NIG (Fig. 4a, b). PhA also increased significantly in the IG (interaction *p* < 0.01), indicating enhanced muscle quality despite minimal within-group changes in the NIG. HbA1c decreased significantly in the IG relative to the NIG (interaction *p* < 0.01). However, the between-group change in eGFR over the 6-month period was not statistically significant (*p* = 0.776), and the interaction between group and time for the annualized change rate (eGFR slope) was also not significant (*p* = 0.559). These secondary outcome results are presented in Table 2 and should be interpreted cautiously as exploratory findings without formal adjustment for multiple comparisons.

**Table 2:** Adjusted Changes in Laboratory and Metabolic Parameters After 6 Months

Outcome	Group	Baseline mean ± SD	Adjusted six months mean ± SD	Between-group difference in change	95% CI	*p* for group × time
HbA1c (%)	IG (n = 25)	7.3 ± 0.6	7.12 ± 0.15	− 0.89	− 1.53 to − 0.25	0.008**
	NIG (n = 20)	7.7 ± 0.9	7.29 ± 0.17	–	–	–
eGFR (mL/min/1.73 m^2^)	IG	58.7 ± 17.5	60.7 ± 7.7	0.65	− 3.84 to 5.14	0.776
	NIG	56.1 ± 17.7	54.6 ± 8.0	–	–	–
Phase Angle (°)	IG	4.45 ± 0.85	4.68 ± 0.21	0.44	0.11 to 0.77	0.01*
	NIG	4.03 ± 1.09	3.95 ± 0.23	–	–	–
Skeletal Muscle Index (kg/m^2^)	IG	7.99 ± 1.42	8.25 ± 0.26	0.37	0.13 to 0.62	0.004**
	NIG	8.14 ± 0.81	8.11 ± 0.29	–	–	–

Data are shown as mean ± standard deviation (SD) or mean change with 95% confidence intervals (CI). Between-group difference in change indicates the adjusted between-group difference in change derived from the linear mixed-effects model. DM, diabetes mellitus; IG, intervention group; NIG, non-intervention group. *P* values were obtained using the linear mixed-effects model for the group × time interaction, adjusted for age, baseline 6MWD, HbA1c, eGFR, and cardiovascular disease. *p < 0.05; **p < 0.01

No changes in antidiabetic or renoprotective medications were made during the study period. No adverse events related to the exercise intervention or wearable device use were observed during the 6-month follow-up in either group, indicating that the intervention was safe and well tolerated. Specifically, there were no episodes of severe hypoglycemia requiring medical assistance, no falls resulting in injury, no musculoskeletal injuries leading to withdrawal, and no cardiovascular events attributable to the intervention. Adherence to the intervention was high: 20 of the 25 participants in the intervention group (80%) maintained a physiotherapist-guided exercise routine at least three times per week, demonstrating good feasibility and adherence in an outpatient setting.

## Discussion

This study provides novel evidence that a physiotherapist-led hybrid intervention integrating wearable device feedback significantly improves exercise capacity, muscle strength, and renal parameters in patients with type 2 diabetes, including those with DKD. The IG also achieved significant improvements in SMI and HbA1c, suggesting benefits for both metabolic. To our knowledge, this is among the first studies to integrate real-time wearable feedback with direct physiotherapist-guided exercise instruction in patients with type 2 diabetes, including those with DKD. This hybrid model, combining digital self-monitoring and professional supervision, represents a novel, pragmatic approach to chronic disease rehabilitation [28–30].

### Mechanisms underlying improvements

Based on the linear mixed-effects model, the between-group difference in change in 6-min walk distance was + 42.4 m (F = 15.0, *p* < 0.001), indicating a statistically significant improvement. Importantly, this magnitude of change falls within the previously reported minimal clinically important difference (MCID) range of 30–50 m, supporting the conclusion that the observed improvement was both statistically and clinically meaningful. Individualized exercise prescriptions based on the FITT principle, combined with continuous activity monitoring, likely contributed to enhanced adherence, with approximately 80% of participants maintaining regular exercise at least three times per week. In addition, exercise-induced increases in skeletal muscle index and phase angle suggest improvements in muscle composition and cellular integrity, potentially reflecting enhanced mitochondrial function and reduced oxidative stress [24, 25]. Structured monitoring and physiotherapist-tailored adjustments to exercise intensity and modality likely supported sustained engagement in exercise therapy while maintaining safety, as no exercise-related adverse events were observed throughout the intervention period.

### Role of wearable devices and physiotherapist engagement

The wrist-worn wearable device enabled continuous activity monitoring and real-time feedback, enhancing self-efficacy and behavioral activation. Monthly physiotherapy sessions reinforced adherence through motivational feedback and individualized goal-setting. Compared with traditional waist-mounted accelerometers, wrist devices show superior adherence and feasibility for long-term monitoring [31, 32]. Such digitally supported, supervised exercise programs can bridge gaps in rehabilitation accessibility, particularly for patients not eligible for formal reimbursement. Previous studies have reported that wearable-guided rehabilitation improves physical activity and cardiovascular outcomes in patients with heart failure and coronary artery disease [16]. The structured monthly physiotherapist interaction may have reinforced behavioral change through motivational interviewing and iterative problem-solving—strategies known to improve adherence in chronic disease management [33].

### Clinical implications

This low-cost, scalable hybrid model may help prevent frailty, reduce cardiovascular complications, and slow CKD progression. Digital feedback reduces time and resource demands, improving feasibility in outpatient settings. Integrating such programs into multidisciplinary diabetes care, potentially via tele-rehabilitation platforms [34], could further expand access and cost-effectiveness. DKD often progresses silently, with acceleration of eGFR decline occurring after albuminuria or moderate renal impairment becomes evident. Clinically, this means that opportunities for early intervention are frequently missed. Targeting patients in earlier disease stages may therefore be particularly meaningful, even when short-term changes in eGFR slope are not detectable [35, 36].

### Limitations

Several limitations should be acknowledged. First, this was a single-center, non-randomized study with a modest sample size, which limits generalizability and allows for potential selection bias related to participant motivation. Although covariate-adjusted mixed-effects models were applied, residual confounding and behavioral changes associated with increased clinical contact (i.e., a Hawthorne effect) cannot be fully excluded. Additionally, the study design did not include a physiotherapist-guided intervention group without wearable devices, which limits the ability to disentangle the independent effect of physiotherapist guidance from wearable-based support.

Second, renal outcomes should be interpreted with caution. No significant between-group difference in eGFR was found after adjustment, indicating no renoprotective effect within this short timeframe. Because serum creatinine is influenced by skeletal muscle mass, the observed increases in SMI may have affected eGFR estimation. Future studies using muscle mass–independent markers such as cystatin C and longer follow-up are needed to clarify renal outcomes. Moreover, all secondary outcomes were analyzed in an exploratory manner without formal multiplicity adjustment, and interpretations should therefore remain cautious to avoid overinterpretation in the context of multiple comparisons.

Third, the wearable device was used primarily as a counseling support tool, and detailed longitudinal physical activity metrics (e.g., step counts, wear time, moderate-to-vigorous physical activity) were not retained for quantitative analysis. Consequently, dose–response relationships between activity levels and clinical outcomes could not be directly examined. Future studies should incorporate standardized protocols for activity monitoring to enable robust evaluation.

Finally, the 6-month follow-up period was insufficient to evaluate long-term sustainability or hard clinical outcomes such as cardiovascular events, dialysis initiation, or mortality. Future multicenter randomized trials with longer follow-up and research-grade activity monitoring are needed to confirm efficacy, clarify mechanisms, and determine long-term clinical impact.

## Conclusion

In outpatients with type 2 diabetes, including those with DKD, physiotherapist-led exercise guidance incorporating wearable feedback improved exercise capacity, muscle quality, and HbA1c, but no short-term renoprotective effect was observed. Wearable-informed physiotherapy may offer a feasible and scalable approach to enhance outpatient exercise therapy.

## Supplementary Information

Below is the link to the electronic supplementary material.

Supplementary file1 (DOCX 25 kb)

## Data Availability

The datasets generated and/or analyzed in the current study are available from the corresponding author upon reasonable request.
