# Supplementary material for: Physiotherapist-guided, wearable-informed exercise improves 6-minute walk distance in patients with type 2 diabetes, including those with diabetic kidney disease: a prospective study
Source: Diabetol Int. 2026 Apr 22;17(3):45. doi: 10.1007/s13340-026-00900-x (PMC13103225; doi:10.1007/s13340-026-00900-x)
Supplement: Supplementary file 1 — Supplementary file1 (DOCX 25 kb) [file 13340_2026_900_MOESM1_ESM.docx]

| Outcome | Group | Adjusted 6 months mean ± SD (m) | Between-group difference in change   (Intervention effect) | 95% CI | *p* for group × time |
| --- | --- | --- | --- | --- | --- |
| 6-minute walk distance (m) | IG (n=25) | 531.4 ± 21.8 | +41.1 | 23.5 to 58.7 | <0.001** |
|  | NIG (n=33) | 423.5 ± 19.6 | – | – | – |
| Weight-bearing index (kgf/kg) | IG | 0.53 ± 0.03 | +0.07 | 0.04 to 0.10 | <0.001** |
|  | NIG | 0.52 ± 0.03 | – | – | – |
| HbA1c (%) | IG | 7.12 ± 0.17 | -0.68 | - 1.19 to - 0.17 | 0.009** |
|  | NIG | 7.30 ± 0.15 | – | – | – |
| Phase Angle (°) | IG | 4.69 ± 0.21 | +0.41 | 0.16 to 0.65 | 0.002** |
|  | NIG | 3.98 ± 0.18 | – | – | – |
| Skeletal Muscle Index (kg/m²) | IG | 8.25 ± 0.25 | +0.32 | 0.11 to 0.52 | 0.003** |
|  | NIG | 8.01 ± 0.23 | – | – | – |

Supplymental Table S1. Intention-to-treat analysis of changes over 6 months

Data are shown as mean ± standard deviation (SD) or mean change with 95% confidence intervals (CI). Between-group difference in change indicates the adjusted between-group difference in change derived from the linear mixed-effects model. Analyses were conducted in the intention-to-treat population, including all allocated participants. P values were obtained using the linear mixed-effects model for the group × time interaction, adjusted for age, baseline 6MWD, HbA1c, eGFR, and cardiovascular disease. IG, intervention group; NIG, non-intervention group. *p < 0.05; **p < 0.01.
